# Highly Important Discovery

**Published:** 1886-11

**Authors:** 


HIGHLY IMPORTANT DISCOVERY.—The London *Lancet* states that Mr. Cresswell Henett has discovered a process by which quinine can be made by synthesis at a cost of three pence an ounce. It was suggested in 1869 by the late Dr. Mattheson of St. Bartholomew Hospital, and Dr. Parker of Netley afterwards rendered aid by his advice, so that the drug can now be manufactured from an article found in any part of the world at little cost by Mr. Henett's process.

---

WITHIN the past two months, according to *Le Journ. de Med. de Paris*, six more of Pasteur's patients have died of rabies. The most remarkable fact is that several persons bitten by the same dogs as were those who placed themselves in Pasteur's care, were no attacked with hydrophobia, while some of those treated by him died of the disease. French journals are now wondering whether the fatal cases were derived from the dogs or from Pasteur's virus.

